# A Pilot Study on Blood Concentration of β-Amyloid (40 and 42) and Phospho-Tau 181 in Horses

**DOI:** 10.3390/vetsci12070610

**Published:** 2025-06-23

**Authors:** Valentina Gazzano, Maria Claudia Curadi, Simona Capsoni, Paolo Baragli, Witold Kêdzierski, Francesca Cecchi, Angelo Gazzano

**Affiliations:** 1 Department of Veterinary Sciences, University of Pisa, 56126 Pisa, Italy; valentina.gazzano@unipi.it (V.G.); maria.claudia.curadi@unipi.it (M.C.C.); paolo.baragli@unipi.it (P.B.); angelo.gazzano@unipi.it (A.G.); 2 Department Neuroscience & Rehabilitation, University of Ferrara, 44124 Ferrara, Italy; simona.capsoni@unife.it; 3 Bio@SNS Laboratory of Biology, Scuola Normale Superiore, 56127 Pisa, Italy; 4 Department of Biochemistry, University of Life Sciences, 20-950 Lublin, Poland; kedzierskiwitold@yahoo.co.uk

**Keywords:** equine, cognitive degeneration, amyloid

## Abstract

In humans, aging is often accompanied by cognitive decline, as seen in Alzheimer’s disease. In contrast, the aging process in horses remains poorly characterized. This study aims to explore the presence of blood-based biomarkers associated with cognitive degeneration in this species. Twenty-three Arabian horses were enrolled, and 5 mL of blood was collected from each to measure serum levels of β-amyloid peptides (Aβ40 and Aβ42) and phosphorylated tau protein (pTau181), both considered reliable indicators of cognitive impairment in other species. Aβ42 was undetectable in all samples, while pTau181 ranged from 5.38 to 54.42 pg/mL and Aβ40 from 67.4 to 743.9 pg/mL. Statistical analysis of the data, performed with the non-parametric Spearman test, did not reveal any correlation between age and the concentrations of Aβ40 and pTau. The pTau/Aβ40 ratio also did not appear to be correlated with the age of the subjects. Interestingly, none of the horses exhibited behavioral changes or clinical signs suggestive of cognitive dysfunction. This absence of symptoms may be related to the undetectable levels of Aβ42, the isoform considered crucial in initiating tau phosphorylation and subsequent neurodegeneration, despite possibly being present at concentrations higher than those typically found in healthy humans.

## Simple Summary

Increased life expectancy is a trend observed not only in humans but also in horses. In people, aging is often associated with cognitive deterioration, such as Alzheimer’s disease, which shares similarities with age-related cognitive decline in dogs. However, the aging process in horses remains largely unexplored. This study aims to investigate the presence of blood biomarkers linked to cognitive degeneration in horses. A total of 23 Arabian horses were examined, and serum levels of β-amyloid peptides (Aβ40 and Aβ42) and phosphorylated tau protein (pTau181) were measured, molecules recognized as reliable indicators of cognitive decline in other species. Notably, Aβ42 was undetectable in all samples. While no correlation was found between age and either Aβ40 or pTau181 levels, a significant positive correlation emerged between Aβ40 and pTau181 concentrations. Interestingly, none of the horses showed behavioral changes or clinical signs suggestive of cognitive dysfunction. This absence of symptoms may be attributed to the undetectable levels of Aβ42, the isoform considered key in triggering cognitive degeneration through tau phosphorylation, even if potentially present at higher levels than those typically found in healthy humans.

## 1. Introduction

Alzheimer’s disease (AD) has emerged in recent years as an increasingly widespread disorder, largely attributable to the progressive extension of human life expectancy [1]. This pathology has severe consequences for humans, including significant reductions in cognitive abilities and the loss of social relationships, even with close family members [2].

The increase in life expectancy is a phenomenon that has been observed also in domestic animals, especially those regarded as pets [3]. Among these, dogs and cats stand out as species experiencing considerable lifespan extensions. Dogs, in particular, have become a promising research model for studying AD, as they display a syndrome of cognitive decline in old age that closely resembles the human condition [4,5,6].

In the equine species, a similar trend of increasing life expectancy has been noted. Many horse owners now regard their animals as pets and totally include them as family members [7]. This growing sensitivity towards equines has fostered interest in improving the management of horses that often exceed 25 years of age, including understanding whether, during aging, they retain their learning and memory capabilities [8].

The defining histopathological features of AD are the extracellular buildup of amyloid-β (Aβ) peptides forming senile plaques, and the intracellular accumulation of neurofibrillary tangles (NFTs) composed of hyperphosphorylated tau protein. A growing body of evidence indicates that, in humans, cerebral Aβ deposition begins many years, often decades, before the clinical symptoms of AD appear and precedes the widespread cortical propagation of tau pathology [9]. Despite this, the precise role of Aβ, ranging from impaired proteostasis to synaptic dysfunction, remains incompletely understood. Likewise, although tau hyperphosphorylation is widely recognized as a key contributor to neuronal death, it is still unclear whether it serves as a driving force or a downstream effect in the disease process.

Among the various theories proposed, the amyloid cascade hypothesis (ACH) seems to remain the most comprehensive model addressing the complex etiology of AD, despite ongoing debate. According to ACH, the accumulation of Aβ initiates a cascade of pathological events, including tau hyperphosphorylation and tangle formation, which subsequently lead to neuroinflammation, synaptic deficits, neuronal degeneration, and ultimately, cognitive impairment and behavioral disturbances [10,11].

It is also recognized that certain factors may independently influence or initiate abnormalities in both Aβ and tau proteins [12]. This concept is known as the “dual pathway hypothesis,” which proposes that shared upstream mechanisms can simultaneously trigger both Aβ accumulation and tau dysfunction [12]. In support of this theory, evidence indicates that while Aβ is required for the development of tau pathology, it is not sufficient on its own. In fact, mouse models that develop Aβ plaques but do not overexpress tau require the introduction of misfolded AD-related tau seeds to exhibit the three hallmark forms of tau pathology seen in Alzheimer’s disease: tau-positive dystrophic neurites surrounding Aβ plaques (NP tau), neuropil threads, and neurofibrillary tangles (NFTs). These distinct tau pathologies emerge at different times and have unique impacts on neural function and behavior [13].

Recent research in human beings has been directed in different directions, to identify possible therapies [14] but also to recognize early markers of AD [15], easy and quick to determine in a laboratory.

In human medicine, particular attention is paid to blood sampling, being minimally invasive, flexible (collection is feasible at home or in the community), and allowing time and cost savings [16]. Moreover, retrospective analyses can be performed on frozen blood and the scalability and accessibility of blood sampling is ideal for large-scale clinical use, as well as for observational and interventional studies [17]. Blood biomarker use is also likely to increase enrolment and retention in population-based and clinic-based studies and expand participant diversity [18]. Such studies would be expected to provide new information on the biological basis of dementia and associated risk factors, with clinical and public health implications [18]. Finally, in clinical trials, blood biomarkers could be used for prescreening, to select initial cohorts for further assessment with CSF or PET biomarkers [18].

Notably, β amyloids (Aβ) [19] and phospho-tau protein181 (pTau181) [20] have been identified in humans as blood markers of AD.

Senile plaques composed of β amyloid peptides are widely recognized as a defining characteristic of AD pathogenesis [21]. β-amyloid peptides have taken center stage in AD research due to genetic studies linking them to all known familial forms of the disease [22]. Aβ is produced via proteolytic cleavage of the Amyloid precursor protein (APP), which is localized to several cellular compartments, including the plasma membrane, trans-Golgi network, endoplasmic reticulum, and mitochondrial membranes. Amyloid precursor protein (APP) is a type I transmembrane protein composed of an extracellular domain, a hydrophobic region that spans the membrane, and a short intracellular tail [23]. Under normal physiological conditions, its soluble form (sAPP) plays a crucial role in promoting neurite extension, synapse formation, synaptic plasticity, and neuronal survival. These functions are mediated, in part, by modulating N-methyl-D-aspartate (NMDA) and gamma-aminobutyric acid (GABA) receptors, thereby contributing to the regulation of intracellular calcium levels and maintaining cellular homeostasis [24,25].

APP can be cleaved by a combination of different secretase complexes, following two pathways: non-amyloidogenic or amyloidogenic. This last process is initiated by β-secretase with the releasing of sAPPα from which γ-secretase generates Aβ peptides with different C-terminal residues. The two primary forms of Aβ (Aβ40 and Aβ42) differ in length, with Aβ40 being more abundant under normal conditions [26]. In fact, about 5–15% of the total Aβ pool is Aβ42, and smaller amounts of other Aβs, both longer and shorter, may be observed [27].

In the course of normal human aging, levels of Aβ peptides (Aβ40 and Aβ42) gradually rise over time in a slow and progressive manner [28]. Although amyloid deposits do not correlate with either disease progression or cognitive decline [29], their spatial and temporal emergence are well described [30]. In the first stage, amyloid deposits occur in the frontal, temporal, and occipital lobes of the neocortex. In the second stage, they spread to all the neocortical association areas, except for primary sensory areas and motor fields. The final stage is characterized by depositions in primary neocortical areas and progressive spreading to the striatum, thalamus, and hypothalamus.

In AD, early and progressive deposition of Aβ42 results in a decline of its level in CSF and plasma. The Aβ42/Aβ40 ratio is considered a more reliable marker for risk assessment and diagnosis. Studies in transgenic mice have shown that increased Aβ40 levels inhibit Aβ42-related amyloidosis and associated mortality [31].

In dogs, extensive research has demonstrated an age-dependent increase in plasma Aβ40 and Aβ42 concentrations in healthy individuals. In contrast, dogs affected by canine cognitive dysfunction syndrome (CDS) show significantly reduced plasma Aβ40 and Aβ42 levels compared to cognitively intact, age-matched controls [32]. Cognitive decline in dogs is further linked to tau synaptic impairment and neuroinflammation [33] literature on canine cognitive dysfunction has led to the development and validation of several clinical rating scales aimed at early and objective assessment of age-related cognitive decline in dogs [34]. These tools, which include both owner-based questionnaires and clinician-administered evaluations, may offer a valuable framework for future adaptation to equine species, where structured cognitive assessment methods are currently lacking.

In the adult human brain, tau protein predominantly resides in axons, where it binds to tubulin promoting polymerization, regulating microtubule stability, and determining microtubule spacing [35]. However, increased tau phosphorylation reduces its affinity for microtubules, causing detachment and dislocation of tau molecules that affect the stability of the microtubule network and may perturb axonal transport processes [36]. In AD, the pathological form of hyperphosphorylated tau (pTau) constitutes the major component of paired helical filaments, which aggregate into neurofibrillary tangles, one of the hallmark neuropathological features of the disease [35].

In humans, plasma pTau181 at baseline is a strong predictor of progression to cognitive decline, which is seen to be comparable to CSF pTau181. In the study by Janelidze et al. [37], it was found that individuals who had abnormal baseline levels of pTau181 had a substantially increased risk of developing AD dementia in the future (HR = 10.9, 95% CI = 5.0–24.0).

In horses, studies on cognitive aging, and its causes, are completely absent, except for some studies conducted on memory [8,38,39,40,41].

The aim of this research is to determine, for the first time in the equine species, the concentrations in the blood serum of Aβ40, Aβ42, and pTau181. We also tried to identify potential correlation between Aβ40, Aβ42, and pTau181 and between these substances and the age of the animals. 

## 2. Materials and Methods

The study included 23 clinically healthy Arabian pureblood horses (19 mares and 4 stallions) aged 1.5 to 24 years (mean ± SD: 10.63 ± 6.4 years) from the “Il Melograno” breeding farm (Pistoia, Tuscany). The number of subjects enrolled was determined through a priori power analysis using G*Power 3.1.9.7 (α = 0.05; 1 − β = 0.80; Cohen’s d = 0.54).

To reduce variability caused by different activities of the animals, which may impact the aging process in horses, only one breed was used. The choice of Arabian horses was based on a specific breeding facility known to us for the high level of care the owners dedicate to monitoring animal health and welfare.

No behavioral changes were reported by the staff and/or the farm veterinarian.

From each horse, a 5 mL blood sample was collected from the jugular vein and kept refrigerated until arrival at the laboratory. There, the samples were centrifuged using an ALC 4237R refrigerated centrifuge (ALC International S.r.l., Milan, Italy) for 15 min at 4 °C. The resulting serum was then stored at −20 °C until analysis. Serum levels of β-amyloid 40, β-amyloid 42, and pTau 181 were subsequently measured using ELISA kits, respectively.

Horse Amyloid Beta Protein 40 (Cat. No: MBS022183), Horse Amyloid Beta Peptide 1-42 (Cat. No: MBS06794), and Horse Phosphorylated Tau 181 (Cat. No: MBS9905651) ELISA kits were purchased from MyBioSource (San Diego, CA 92195-3308, USA).

Statistical analysis was performed using Jamovi (version 2.5), retrieved from https://www.jamovi.org (The Jamovi Project, 2024), accessed on 25 April 2025. Spearman’s rank correlation test was used after verifying, with the Shapiro–Wilk test, that the data were not normally distributed. All statistical analyses were conducted with a significance level set at *p* < 0.05.

## 3. Results

Table 1 presents the sex and age of the horses included in the study, along with the serum concentrations of Aβ40 (pg/mL), pTau181 (pg/mL), and their ratio (pTau181/Aβ40). Aβ42 concentrations were below the detection limit in all samples. Serum pTau181 levels ranged from 5.38 to 54.42 pg/mL, while Aβ40 concentrations ranged from 66.7 to 776 pg/mL.

Statistical analysis of the data, performed with the non-parametric Spearman test, did not reveal any correlation between age and the concentrations of Aβ40 and pTau. The pTau/Aβ40 ratio also did not appear to be correlated with the age of the subjects.

**Figure 1:**
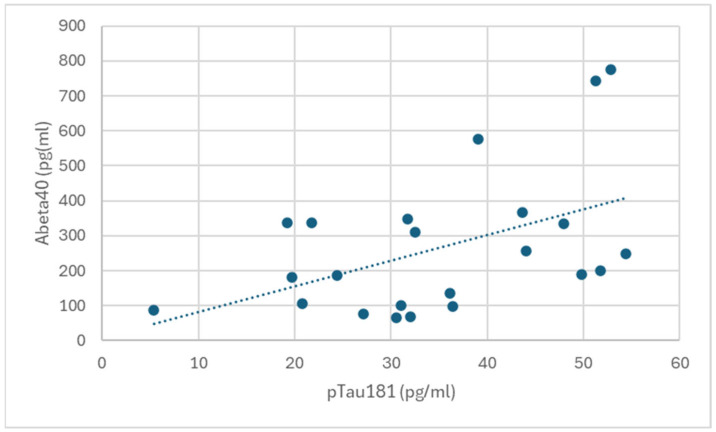
Positive and statistically significant correlation between blood concentrations of Aβ40 and pTau (Rho = 0.453; *p* = 0.017).

The presence, instead, of a positive correlation, statistically significant (Rho = 0.453; *p* = 0.017) was found between the blood concentrations of Aβ40 and pTau, as shown in Figure 1.

## 4. Discussion

The progressive increase in equine life expectancy raises the critical question of how to ensure successful aging in this species [42]. While numerous studies in dogs have led to the development of cognitive evaluation tools [34], research on cognitive decline in horses remains limited, and no data are currently available regarding potential markers of neuronal degeneration in this species. To our knowledge, the findings presented here represent the first published data on this topic in horses and constitute a crucial step toward identifying the paraphysiological factors involved in equine cognitive decline.

The data obtained reveals the presence of the same substances identified in aging humans and dogs, except for Aβ42. However, the limited sample size in this study precludes definitive conclusions and further studies on older horses will be necessary to understand whether β amyloids and tau protein circulating in the blood may be somehow related to cerebral senescence, as demonstrated in other species [32,33].

New, more sensitive analysis techniques will also need to be applied, which allow lower concentrations of these substances to be detected. In this regard, the Single-Molecule Array (SIMOA^®^) technique, an ultrasensitive single-molecule array, represents a novel, highly sensitive platform that can detect thousands of single molecules simultaneously [43]. Unlike traditional ELISA systems, which require a high number of enzyme labels to generate reader-detectable signals, SIMOA ^®^ employs femtoliter-sized reaction chambers that can isolate and detect single enzyme molecules.

In dogs, SIMOA ^®^ has successfully measured blood concentrations of Aβ40 and Aβ42 [32]. In our study, the inability to detect serum levels of Aβ42 might be due to the relatively low sensitivity of the ELISA technique, which is approximately a thousand times less powerful than SIMOA ^®^.

We cannot exclude, based on current knowledge, that in the equine species there is a different processing of APP that does not lead to the formation of Aβ42 or that its production is so low that it is not detectable. Further studies with more advanced analytical techniques will be necessary to verify this possibility.

Interestingly, our analysis revealed a positive correlation between Aβ40 and pTau181 protein concentrations. This is the first report suggesting a possible relationship between these two substances in horses, necessitating further research to establish whether a causal link exists. All vertebrate species generate APP and β-secretase: in birds, reptiles, and amphibians Aβ shares more than 90% of its amino acid sequence with the human form, while in mammals, the similarity exceeds 95% [44]. The high degree of sequence conservation across vertebrate evolution suggests that Aβ provides a survival benefit. This idea is further supported by findings showing that reducing natural levels of Aβ leads to negative effects in multiple species and experimental models [45].

The increased presence of Aβ40 may not always indicate pathology; for instance, elevated Aβ40 levels have been observed in aging dogs without cognitive impairments [32]. A growing body of scientific literature indicates that, under certain physiological conditions, amyloid-β (Aβ) can exert neuroprotective and trophic effects [46,47]. Although these functions may appear to conflict with Aβ’s well-known neurotoxic role in pathological contexts [48], evidence suggests that these opposing properties are not mutually exclusive but rather depend on specific biological conditions [49]. Aβ40 has been shown to promote membrane cholesterol dynamics and availability, contributing to the structural and functional integrity of neuronal membranes [50], thereby reinforcing the idea that Aβ, under non-pathological conditions, may serve important homeostatic roles in the brain. Supporting this idea, an in vivo study in rats demonstrated that blocking endogenous Aβ through the hippocampal infusion of a monoclonal antibody targeting its ectodomain, administered immediately before a learning task, significantly impaired both short- and long-term memory retention in an inhibitory avoidance test. Notably, memory was unaffected when the same antibody was administered after the training session, suggesting a functional role for Aβ in memory consolidation [51].

Tau protein phosphorylation is instead considered pathological, as pTau181 is a major component of paired helical filaments and neurofibrillary tangles [35]. It is important to mention that while Aβ accumulation is characteristic of AD, tau pathology also exists in a group of neurodegenerative diseases known as tauopathies, which are different from [52].

The mechanisms behind Aβ and tau interplay in AD remain elusive; nevertheless, currently, it is becoming widely accepted that Aβ (especially Aβ42) is the “trigger” and tau the “bullet” driving AD [53]. In addition, it has recently been reported that the presence of both Aβ and tau is necessary for memory decline in the preclinical stages of AD [54]. For this reason, it is of fundamental importance to know the relationships that exist between these two proteins at the blood level since they could reveal themselves, also for the equine species, as prognostic markers of cerebral degeneration.

Interestingly, none of the animals in our study exhibited behavioral alterations or clinical signs indicative of cognitive decline (e.g., standing motionless in a corner of the stall, sleep–wake cycle disturbances, aimless wandering, failure to recall learned tasks, or aggressive behavior). One possible explanation is that the absence of such signs may be related to undetectable levels of Aβ42, the specific isoform believed to initiate cognitive degeneration through tau protein phosphorylation. Although pTau181 may be present at concentrations higher (39.94 ± 13.09 pg/mL, mean value ± SD) than those typically found in healthy humans (8.8 ± 10.1 pg/mL, mean value ± SD) [55], its levels in these animals might still fall below the threshold required to trigger pathological processes.

Assessing cognitive dysfunction in horses remains a significant challenge, primarily due to the absence of validated diagnostic criteria and cognitive assessment scales in equine medicine. Unlike dogs, which typically live in close daily contact with humans, making owner-based questionnaires feasible, horses are managed in entirely different contexts, often limiting the collection of reliable behavioral data. This diagnostic gap complicates the ability to determine whether animals are truly unaffected by cognitive decline and highlights the urgent need to develop specific and standardized behavioral tests tailored to equine species.

Despite these limitations, this pilot study presents several strengths. It represents, to our knowledge, the first attempt to investigate in horses the presence of circulating substances, already studied in humans and dogs, as potential blood-based biomarkers of cognitive degeneration. However, several critical constraints must be acknowledged. The small sample size, particularly the low number of geriatric animals, limits the generalizability of the findings. Furthermore, we cannot exclude the possibility that Aβ42 levels were undetectable simply because none of the animals had developed cognitive dysfunction. The use of conventional analytical techniques may also have limited the detection of Aβ42 in serum samples. Finally, the lack of validated cognitive assessment scales for the equine species prevents any correlation between the severity of symptoms and the animal’s behavior.

These limitations underscore the necessity for further research employing more advanced methodologies and larger, age-stratified cohorts. Future studies should aim to clarify whether the observed positive correlation between Aβ40 and pTau181 has prognostic value in assessing the severity of cognitive decline in horses, and whether reduced Aβ42 levels are insufficient to trigger tau protein phosphorylation.

## 5. Conclusions

This pilot study constitutes a preliminary step toward a systematic investigation of brain aging in the equine species. The identification of key molecules implicated in neurodegeneration provides a foundation for future research, which may employ more advanced analytical techniques to explore potential correlations supporting the use of these substances as biomarkers of cognitive decline.

## Figures and Tables

**Table 1:** Sex, age, and serum concentration of Aβ40 (pg/mL), pTau (pg/mL), and their ratio (pTau181/Aβ40) in the horses included in the study.

	Sex	Age (Years)	pTau (pg/mL)	Aβ40 (pg/mL)	pTau/Aβ40 Ratio
1	Mare	1.5	32.05	67.4	0.47
2	Mare	3	52.82	776.0	0.07
3	Mare	4	36.41	98.9	0.37
4	Mare	4	19.74	181.0	0.10
5	Mare	4	43.65	366.0	0.12
6	Mare	5	44.04	256.0	0.17
7	Mare	6	24.36	186.0	0.13
8	Mare	6	49.81	188.9	0.26
9	Stallion	8	51.28	743.9	0.07
10	Mare	9	5.38	86.0	0.06
11	Mare	9	19.23	338.1	0.06
12	Mare	9	51.73	198.9	0.26
13	Mare	11	31.03	101.7	0.30
14	Mare	12	36.15	135.3	0.26
15	Stallion	12	27.18	77.4	0.35
16	Mare	13	32.5	309.6	0.10
17	Mare	14	47.95	333.9	0.14
18	Mare	14	20.77	104.6	0.19
19	Mare	15	31.73	346.7	0.09
20	Mare	18	21.79	388.1	0.06
21	Stallion	21	30.58	66.7	0.46
22	Mare	22	54.42	249.6	0.22
23	Stallion	24	39.04	575.3	0.07

## Data Availability

All data generated or analyzed during this study are included in the manuscript.

## References

[1] Zhang X., Guo T., Zhang Y., Jiao M., Ji L., Dong Z., Li H., Chen S., Zheng W., Jing Q. (2024). Global Burden of Alzheimer’s Disease and Other Dementias Attributed to Metabolic Risks from 1990 to 2021: Results from the Global Burden of Disease Study 2021. BMC Psychiatry.

[2] Mohamed S., Rosenheck R., Lyketsos C.G., Schneider L.S. (2010). Caregiver Burden in Alzheimer Disease: Cross-Sectional and Longitudinal Patient Correlates. Am. J. Geriatr. Psychiatry.

[3] Ciurli L., Casini L., Cecchi F., Baragli P., Macchioni F., Curadi C., Gazzano V., Capsoni S., Gazzano A. (2023). The Canine Cognitive Dysfunction Syndrome: Epidemiology, Pathophysiology and Diagnosis. Dog Behav..

[4] Bosch M.N., Pugliese M., Gimeno-Bayón J., Rodríguez M.J., Mahy N. (2012). Dogs with Cognitive Dysfunction Syndrome: A Natural Model of Alzheimer’s Disease. Curr. Alzheimer Res..

[5] Osella M.C., Re G., Odore R., Girardi C., Badino P., Barbero R., Bergamasco L. (2007). Canine Cognitive Dysfunction Syndrome: Prevalence, Clinical Signs and Treatment with a Neuroprotective Nutraceutical. Appl. Anim. Behav. Sci..

[6] Seisdedos Benzal A., Galán Rodríguez A. (2016). Recent Developments in Canine Cognitive Dysfunction Syndrome. Pet. Behav. Sci..

[7] American Veterinary Medical Association (2007). Pet Ownership & Demographics Sourcebook.

[8] Cellai S., Gazzano A., Casini L., Gazzano V., Cecchi F., Macchioni F., Cozzi A., Pageat L., Arroub S., Fratini S. (2024). The Memory Abilities of the Elderly Horse. Animals.

[9] Roda A., Serra-Mir G., Montoliu-Gaya L., Tiessler L., Villegas S. (2022). Amyloid-Beta Peptide and Tau Protein Crosstalk in Alzheimer’s Disease. Neural Regen. Res..

[10] Karran E., De Strooper B. (2016). The Amyloid Cascade Hypothesis: Are We Poised for Success or Failure?. J. Neurochem..

[11] Selkoe D.J., Hardy J. (2016). The Amyloid Hypothesis of Alzheimer’s Disease at 25 Years. EMBO Mol. Med..

[12] Small S.A., Duff K. (2008). Linking Aβ and Tau in Late-Onset Alzheimer’s Disease: A Dual Pathway Hypothesis. Neuron.

[13] He Z., Guo J.L., McBride J.D., Narasimhan S., Kim H., Changolkar L., Zhang B., Gathagan R.J., Yue C., Dengler C. (2018). Amyloid-β Plaques Enhance Alzheimer’s Brain Tau-Seeded Pathologies by Facilitating Neuritic Plaque Tau Aggregation. Nat. Med..

[14] Elhage A., Cohen S., Cummings J., van der Flier W.M., Aisen P., Cho M., Bell J., Hampel H. (2024). Defining Benefit: Clinically and Biologically Meaningful Outcomes in the next-Generation Alzheimer’s Disease Clinical Care Pathway. Alzheimer’s Dement..

[15] Therriault J., Schindler S.E., Salvadó G., Pascoal T.A., Benedet A.L., Ashton N.J., Karikari T.K., Apostolova L., Murray M.E., Verberk I. (2024). Biomarker-Based Staging of Alzheimer Disease: Rationale and Clinical Applications. Nat. Rev. Neurol..

[16] Karikari T.K., Ashton N.J., Brinkmalm G., Brum W.S., Benedet A.L., Montoliu-Gaya L., Lantero-Rodriguez J., Pascoal T.A., Suárez-Calvet M., Rosa-Neto P. (2022). Blood Phospho-Tau in Alzheimer Disease: Analysis, Interpretation, and Clinical Utility. Nat. Rev. Neurol..

[17] Hampel H., O’Bryant S.E., Molinuevo J.L., Zetterberg H., Masters C.L., Lista S., Kiddle S.J., Batrla R., Blennow K. (2018). Blood-Based Biomarkers for Alzheimer Disease: Mapping the Road to the Clinic. Nat. Rev. Neurol..

[18] Ashton N.J., Hye A., Rajkumar A.P., Leuzy A., Snowden S., Suárez-Calvet M., Karikari T.K., Schöll M., La Joie R., Rabinovici G.D. (2020). An Update on Blood-Based Biomarkers for Non-Alzheimer Neurodegenerative Disorders. Nat. Rev. Neurol..

[19] Blennow K., Mattsson N., Schöll M., Hansson O., Zetterberg H. (2015). Amyloid Biomarkers in Alzheimer’s Disease. Trends Pharmacol. Sci..

[20] Hampel H., Blennow K., Shaw L.M., Hoessler Y.C., Zetterberg H., Trojanowski J.Q. (2010). Total and Phosphorylated Tau Protein as Biological Markers of Alzheimer’s Disease. Exp. Gerontol..

[21] Hardy J., Selkoe D.J. (2002). The Amyloid Hypothesis of Alzheimer’s Disease: Progress and Problems on the Road to Therapeutics. Science.

[22] Selkoe D.J. (2000). The genetics and molecular pathology of alzheimer’s disease roles of amyloid and the presenilins. Neurol. Clin..

[23] Kang J., Lemaire H.G., Unterbeck A., Salbaum J.M., Masters C.L., Grzeschik K.H., Multhaup G., Beyreuther K., Müller-Hill B. (1987). The precursor of Alzheimer’s disease amyloid A4 protein resembles a cell-surface receptor. Nature.

[24] Müller U.C., Deller T., Korte M. (2017). Not Just Amyloid: Physiological Functions of the Amyloid Precursor Protein Family. Nat. Rev. Neurosci..

[25] Rice H.C., De Malmazet D., Schreurs A., Frere S., Van Molle I., Volkov A.N., Creemers E., Vertkin I., Nys J., Ranaivoson F.M. (2019). Secreted Amyloid-b Precursor Protein Functions as a GABA B R1a Ligand to Modulate Synaptic Transmission. Science.

[26] Younkin S.G. (1998). The Role of Aβ42 in Alzheimer’s Disease. J. Physiol. Paris.

[27] Teng F.Y.H., Tang B.L. (2005). Widespread γ-Secretase Activity in the Cell, but Do We Need It at the Mitochondria?. Biochem. Biophys. Res. Commun..

[28] Thorwald M.A., Silva J., Head E., Finch C.E. (2023). Amyloid Futures in the Expanding Pathology of Brain Aging and Dementia. Alzheimer’s Dement..

[29] Hanseeuw B.J., Betensky R.A., Jacobs H.I.L., Schultz A.P., Sepulcre J., Becker J.A., Cosio D.M.O., Farrell M., Quiroz Y.T., Mormino E.C. (2019). Association of Amyloid and Tau with Cognition in Preclinical Alzheimer Disease: A Longitudinal Study. JAMA Neurol..

[30] Braak H., Braak E. (1991). Neuropathological Stageing of Alzheimer-Related Changes. Acta Neuropathol..

[31] Kim J., Onstead L., Randle S., Price R., Smithson L., Zwizinski C., Dickson D.W., Golde T., McGowan E. (2007). Aβ40 Inhibits Amyloid Deposition in Vivo. J. Neurosci..

[32] Panek W.K., Murdoch D.M., Gruen M.E., Mowat F.M., Marek R.D., Olby N.J. (2021). Plasma Amyloid Beta Concentrations in Aged and Cognitively Impaired Pet Dogs. Mol. Neurobiol..

[33] Smolek T., Madari A., Farbakova J., Kandrac O., Jadhav S., Cente M., Brezovakova V., Novak M., Zilka N. (2016). Tau Hyperphosphorylation in Synaptosomes and Neuroinflammation Are Associated with Canine Cognitive Impairment. J. Comp. Neurol..

[34] Ciurli L., Casini L., Cecchi F., Baragli P., Macchioni F., Curadi M.C., Gazzano V., Capsoni S., Gazzano A. (2023). The Canine Cognitive Dysfunction Syndrome: Rating Scales. Dog Behav..

[35] Iqbal K. (2024). Tau and Alzheimer’s Disease: Past, Present and Future. Cytoskeleton.

[36] Arendt T., Stieler J.T., Holzer M. (2016). Tau and Tauopathies. Brain Res. Bull..

[37] Janelidze S., Mattsson N., Palmqvist S., Smith R., Beach T.G., Serrano G.E., Chai X., Proctor N.K., Eichenlaub U., Zetterberg H. (2020). Plasma P-Tau181 in Alzheimer’s Disease: Relationship to Other Biomarkers, Differential Diagnosis, Neuropathology and Longitudinal Progression to Alzheimer’s Dementia. Nat. Med..

[38] McLean A.N. (2004). Short-Term Spatial Memory in the Domestic Horse. Appl. Anim. Behav. Sci..

[39] Hanggi E.B., Ingersoll J.F. (2009). Long-Term Memory for Categories and Concepts in Horses (Equus Caballus). Anim. Cogn..

[40] Wolff A., Hausberger M. (1996). Learning and Memorisation of Two Different Tasks in Horses: The Effects of Age, Sex and Sire. Appl. Anim. Behav. Sci..

[41] Marinier S., Alexander A.J. (1994). The Use of a Maze in Testing Learning and Memory in Horses. Appl. Anim. Behav. Sci..

[42] Ballou M.E., Mueller M.K., Dowling-Guyer S. (2020). Aging Equines: Understanding the Experience of Caring for a Geriatric Horse with a Chronic Condition. J. Equine Vet. Sci..

[43] Dong R., Yi N., Jiang D. (2024). Advances in Single Molecule Arrays (SIMOA) for Ultra-Sensitive Detection of Biomolecules. Talanta.

[44] Tharp W.G., Sarkar I.N. (2013). Origins of Amyloid-β. BMC Genom..

[45] Brothers H.M., Gosztyla M.L., Robinson S.R. (2018). The Physiological Roles of Amyloid-β Peptide Hint at New Ways to Treat Alzheimer’s Disease. Front. Aging Neurosci..

[46] Plant L.D., Boyle J.P., Smith I.F., Peers C., Pearson H.A. (2003). The Production of Amyloid β Peptide Is a Critical Requirement for the Viability of Central Neurons. J. Neurosci..

[47] López-Toledano M.A., Shelanski M.L. (2004). Neurogenic Effect of β-Amyloid Peptide in the Development of Neural Stem Cells. J. Neurosci..

[48] Li Y.Q., Tan S.S., Wu D., Zhang Q., Wang T., Zheng G. (2025). The Role of Intracellular and Extracellular Copper Compartmentalization in Alzheimer’s Disease Pathology and Its Implications for Diagnosis and Therapy. Front. Neurosci..

[49] Carrillo-Mora P., Luna R., Colín-Barenque L. (2014). Amyloid Beta: Multiple Mechanisms of Toxicity and Only Some Protective Effects?. Oxid. Med. Cell Longev..

[50] Koudinov A., Koudinova N. (2003). Amyloid Beta Protein Restores Hippocampal Long Term Potentiation: A Central Role for Cholesterol?. Neurobiol. Lipids.

[51] Puzzo D., Privitera L., Leznik E., Fà M., Staniszewski A., Palmeri A., Arancio O. (2008). Picomolar Amyloid-β Positively Modulates Synaptic Plasticity and Memory in Hippocampus. J. Neurosci..

[52] Zhang Y., Wu K.M., Yang L., Dong Q., Yu J.T. (2022). Tauopathies: New Perspectives and Challenges. Mol. Neurodegener..

[53] Bloom G.S. (2014). Amyloid-β and Tau: The Trigger and Bullet in Alzheimer Disease Pathogenesis. JAMA Neurol..

[54] Sperling R.A., Mormino E.C., Schultz A.P., Betensky R.A., Papp K.V., Amariglio R.E., Hanseeuw B.J., Buckley R., Chhatwal J., Hedden T. (2019). The Impact of Amyloid-Beta and Tau on Prospective Cognitive Decline in Older Individuals. Ann. Neurol..

[55] Zetterberg H., Wilson D., Andreasson U., Minthon L., Blennow K., Randall J., Hansson O. (2013). Plasma Tau Levels in Alzheimer’s Disease. Alzheimers Res. Ther..

